# The Relationship between Expressive Language Sampling and Clinical Measures in Fragile X Syndrome and Typical Development

**DOI:** 10.3390/brainsci10020066

**Published:** 2020-01-26

**Authors:** Rebecca C. Shaffer, Lauren Schmitt, Angela John Thurman, Leonard Abbeduto, Michael Hong, Ernest Pedapati, Kelli Dominick, John Sweeney, Craig Erickson

**Affiliations:** 1 Cincinnati Children’s Hospital Medical Center, Cincinnati, OH 45229, USA; lauren.schmitt@cchmc.org (L.S.); ernest.pedapati@cchmc.org (E.P.); Kelli.Dominick@cchmc.org (K.D.); craig.erickson@cchmc.org (C.E.); 2 Department of Pediatrics, University of Cincinnati College of Medicine, Cincinnati, OH 45229, USA; sweeneyja@hotmail.com; 3 MIND Institute, University of California Davis, Sacramento, CA 95817, USA; ajthurman@ucdavis.edu (A.J.T.); ljabbeduto@ucdavis.edu (L.A.); 4 Department of Psychiatry and Behavioral Sciences, University of California Davis Health, Sacramento, CA 95817, USA; 5 Department of Psychiatry, University of Cincinnati College of Medicine, Cincinnati, OH 45229, USA; hongmp@mail.uc.edu

**Keywords:** Fragile X Syndrome, expressive language sampling, language impairments, ASD, hyperactivity, talkativeness, intelligibility

## Abstract

Language impairment is a core difficulty in fragile X syndrome (FXS), and yet standardized measures lack the sensitivity to assess developmental changes in the nature of these impairments. Expressive Language Sampling Narrative (ELS-N) has emerged as a promising new measure with research demonstrating its usefulness in a wide range of ages in developmental disabilities and typical development. We examined ELS-N results in FXS and age-matched typically developing (TD) controls along with cognitive, adaptive, and clinical measures. We found the groups differed significantly on all ELS-N variables. Cognitive abilities were related to lexical diversity, syntactic complexity, and unintelligibility for the FXS group, but only verbal abilities were related to syntactic complexity in TD. Autism spectrum disorder (ASD) symptomatology was related to less intelligibility in speech. Measures of hyperactivity were related to increased talkativeness and unintelligibility. In addition, FXS males in comparison to FXS females were more impaired in cognitive ability, ASD symptoms, hyperactivity, and anxiety. This study extends the previous ELS research, supporting its use in FXS research as a measure to characterize language abilities. It also demonstrates the relationships between ELS-N variables and measures of cognitive, adaptive, ASD symptoms, and clinical symptoms.

## 1. Introduction

Fragile X syndrome (FXS) is the most common inherited cause of intellectual disability and the most commonly known single-gene cause of autism spectrum disorder. FXS results from a mutation on the *FMR1* gene and an expansion of CGG repeats to 200 or more. This leads to a lack of production of FMRP, the gene’s protein. FMRP is important for learning and memory and its absence leads to the phenotypic characteristics of FXS, including language impairment.

The majority of individuals with FXS have delayed language development, beginning at the prelinguistic period, the time before children say their first meaningful word, and continuing into childhood and adolescence [1]. Delays are present across vocabulary, morphosyntax, and pragmatics. In addition to language delays, individuals with FXS tend to have high rates of verbal perseveration such as repetition of words and topics [2,3]. As with overall functioning, language development tends to be more impaired in males with FXS than females with FXS, and individuals with FXS with co-occurring autism spectrum disorder (ASD) tend to show less intelligible language [4].

FXS has had a very active translation of preclinical to clinical trials, with disappointing results, despite promising animal findings and a strong commitment and level of satisfaction from participants and families. It has been difficult to determine the extent to which this is due to the placebo effect versus outcome measures that lack sensitivity to change. The latter is particularly true in the area of language impairment. In fact, National Institute of Health work groups focused on FXS have identified the need for the development of language outcome measures and placed it as a high priority for researchers. Expressive language sampling (ELS) has been suggested as an appropriate and ecologically valid measure to meet this need in FXS. ELS offers a more naturalistic measurement of language versus typical standardized language assessments. In addition, males with FXS tend to display significant floor effects on many standardized tests, limiting the possibility to obtain a true estimate of ability and to validly assess any change in performance between time points [4]. Clear guidelines for ELS were developed by Abbeduto and team at the MIND Institute [5]. A working group that convened on FXS outcome measurement gave it a “moderate tool quality” and agreed that ELS holds more promise than other available language measures for use in clinical trials [6]. Pilot trials with ELS have demonstrated strong reproducibility and clinical validity [7], and it has been utilized with FXS, Down syndrome (DS), and recently with a typical population [5,7,8].

The ELS administration procedures developed by Kover, McDuffie, Abbeduto and Brown [5], involve both dyadic conversation and narrative sampling techniques. The focus of this paper will be on the narrative sampling component (ELS-N). In ELS-N, participants are presented with wordless picture books (Frog Goes to Dinner [9] or Frog on his Own) [10]. After an initial introduction, the participant tells the story based on the pictures. The auditory recording of the session is then transcribed using conventions commonly used in child language research and analyzed with Systematic Analysis of Language Transcripts (SALT), a computer program that automatically generates several outcome measures from the transcripts, including: communication units (C-units), syntactic complexity (mean length of C-units in morphemes), lexical diversity (number of different word roots used), talkativeness, utterance planning (occurrence of dysfluencies), intelligibility (articulation quality), with other measures possible as well. Kover, McDuffie, Abbeduto and Brown [5] described the structure in further detail.

An early study of the ELS-N measure was completed with adolescents with FXS or DS and typically developing (TD) preschoolers, all matched by nonverbal mental age [5]. Individuals with DS produced the lowest number of C-units, followed by FXS, and finally TD preschoolers. The measure was feasible with all participants. A second pilot of ELS-N extended the age range to males and females with FXS between the ages of 5 and 30 years old [7]. Fidelity was achieved by minimally trained research assistants. Feasibility, good reproducibility, and minimal practice effects were demonstrated for verbal individuals in this broad age range. All participants were able to produce adequate language to evaluate, and there were no subject ceiling or floor effects during testing. Additionally, correlations were found between ELS-N measures and Vineland Adaptive Behavior expressive and written language scores as reported by the parent, suggesting ELS-N not only has sound psychometric properties for use in clinical trials but also may be well-suited to detect smaller changes in language functioning that other standardized measures fail to do.

In addition to utilizing ELS-N with FXS and other developmental disabilities, Channell, Loveall, Conners, Harvey and Abbeduto [8] recently evaluated it in TD individuals aged 4–21 years old. ELS-N was feasible and valid for the entire age range. In cross-sectional analyses, there were age-related increases in syntactic complexity and lexical diversity up to age 18.5, demonstrating that the measure captures developmental performance through adolescence. It also demonstrated that individuals with developmental ages that correspond to the chronological ages of the study—4–21—can perform the task.

The current study was embedded within a larger clinical single-dose pharmaceutical trial through a U54 Centers grant. We focused on the ELS-N data obtained at baseline for participants with FXS enrolled in the study. Our first aim was to further expand validity evidence of the ELS-N by comparing the FXS group with a chronologically age matched TD group. This is the first known study to actively compare results between FXS and same aged controls, as previous studies have independently studied FXS or TD or compared two developmentally delayed groups. We hypothesized that there would be a significant difference between the two groups. Secondly, we aimed to evaluate relationships between ELS-N and standardized assessments of intellectual ability and adaptive behavior in order to evaluate criterion and discriminant validity. To date, studies utilizing ELS have only compared results to other language measures and nonverbal intellectual abilities. We hypothesized that more impaired language abilities, as measured by ELS-N, would be strongly related to both verbal and nonverbal intellectual ability and communication, as measured through parent report on adaptive assessment measures. Finally, exploratory analyses were conducted between ELS-N and measures of mental health and behavior to further examine criterion and discriminant validity. We hypothesized that individuals with more severe language impairments would exhibit higher levels of behavior difficulties. The analyses reported thus, addressing the construct validity of the ELS-N procedure.

## 2. Materials and Methods

All subjects or their guardians gave their informed consent for inclusion before they participated in the study. The study was conducted in accordance with the Declaration of Helsinki, and the protocol was approved by the Ethics Committee of Cincinnati Children’s Hospital Medical Center. Participants with FXS were enrolled in the study if they had genetic results demonstrating >200 CGG repeats. Healthy typically developing controls (TDC) were recruited through web-based fliers from the local community and were matched on sex and age within 4 years to a FXS participant. TDC had no known prior diagnosis of or treatment for developmental or neuropsychiatric disorders. No participant had a history of seizure disorder or current use of anticonvulsant medication, benzodiazepine, or novel potential treatment for FXS (i.e., minocycline). We enrolled 25 individuals with full mutation FXS and 23 age-matched TD controls between the ages of 5 and 36 (FXS M = 19.9 ± 8.4; TD M = 19.2 ± 8.4). All the participants who attempted ELS-N were able to complete and provide adequate utterances to be analyzed. The FXS group had 14 males and 11 females and the TD group had 11 males and 12 females. Of the FXS group, 3 male participants had an additional diagnosis of autism spectrum disorder, which was confirmed via ADOS-2 administration.

Participants completed the assessments for this study in a single visit of the larger study. Participants were administered the ELS-N and the Stanford-Binet 5 Brief IQ by a research coordinator. Parents or caregivers completed the Vineland Adaptive Behavior Scales (2nd edition), Aberrant Behavior Checklist, Anxiety, Depression, and Mood Scale (ADAMS), and the Social Communication Questionnaire (SCQ).

### 2.1. Expressive Language Sampling Narrative (ELS-N)

The procedures outlined by Kover, McDuffie, Abbeduto and Brown [5] were followed for the ELS-N. Participants were asked to tell the story of a wordless picture book “Frog Goes to Dinner.” First the examiner showed the participant each page of the book at a 10 s interval per page. The examiner then asked the participant to tell the story page by page. The examiner utilized scripted prompts to encourage the participants to narrate the book with his/her own words. All samples were gathered at the Cincinnati Fragile X Center by a research coordinator trained in ELS methodology. Digital audio-recorded samples were then de-identified and transferred to the lab of Leonard Abbeduto at the UC Davis MIND Institute for transcription and analysis. Each sample was analyzed by a primary transcriber and then checked by a second transcriber who compared the transcript with the recording. All transcribers were trained to high levels of reliability before initiating transcription, which included reaching agreement with a standard on both the dyadic conversation and narrative sampling techniques (ELS-N), in each of three different participant groups (i.e., TD, FXS, and DS). To achieve reliability, the following criteria were met: (1) >80% agreement in the identification of partly or fully unintelligible C-units, dysfluencies, interruptions/abandonments, instances of overlapping speech, and pauses and (2) >70% agreement for utterance segmentation into C-units as well as the identification of the exact number of words, morphemes, word/morphemic content, and punctuation.

The samples were transcribed using Systematic Analysis of Language Transcripts, 2018 Research Version software (SALT) [11]. The SALT program performs predetermined and customized analysis of text language files. All speech was segmented into C-units (defined as an independent clause and all its modifiers, including dependent clauses) rather than utterances to avoid over-estimating language abilities in highly verbal individuals [12]. Syntactic complexity, lexical diversity, dysfluency, unintelligibility, and talkativeness were computed for all participants. Syntactic complexity was defined as mean length of C-units in morphemes. Lexical diversity, a reflection of range of vocabulary usage, was defined as the number of different word roots in up to 50 C-units. Dysfluency included a proportion of C-units containing mazes (including filled pauses and partial or full repetitions). Unintelligibility was defined as the percentage of C-units that were either partially or completely not understandable during transcription. Finally, talkativeness was assessed based on the number of utterances per minute.

### 2.2. Clinical Measures

Additional measures were administered to the FXS and TD groups to assess a broad range of functioning, behavior, and mental health concerns. Unless indicated, all measures were administered to both the FXS and TD groups. The abbreviated IQ subtests of the Stanford-Binet 5 were administered as a standard measure of cognitive abilities. The Stanford Binet-5 Abbreviated is composed of a verbal and a nonverbal subtest and provides an abbreviated IQ score (ABIQ). Updated deviation scoring procedures for the SB-5 have been created to allow a lower floor of the measure [13]. We utilized these z-scores for the two subscales and the deviation score for the ABIQ. The Social Communication Questionnaire (SCQ) assesses the presence of social communication difficulties related to autism spectrum disorder. The Aberrant Behavior Checklist (ABC) is a 58-item questionnaire with 5 subscales derived by factor analysis: irritability, social withdrawal, stereotypy, hyperactivity, and inappropriate speech. It has been extensively used in psychopharmacological studies of FXS. The Anxiety, Depression, and Mood Scale (ADAMS) comprehensively measures symptoms of anxiety and depression in individuals with developmental and intellectual disabilities [14]. It is comprised of 5 subscales including manic/hyperactivity, depressed mood, social avoidance, general anxiety, and compulsive behavior. The Vineland-II was used to assess adaptive functioning in the FXS group only. It is comprised of four domains: communication, daily living skills, socialization, and motor skills. This is a well-standardized open-ended interview with a primary caregiver used to assess the overall functioning of youth and adults. Standard scores and v-scale scores were utilized for this measure. The Vineland-II was not completed with the TD group.

### 2.3. Statistical Analyses

To test the differences between the FXS and TD groups, a series of ANCOVAs were conducted by testing between-group differences on each of the ELS-N variables. Within group analyses were conducted to examine any sex differences on the ELS-N variables for the FXS and the TD groups. Due to the wide age range, the age of participants was used as a covariate in all analyses. Pearson correlations were computed between psychological assessments and the ELS-N variables to assess any relationships between intelligence, adaptive behavior, mental health concerns, aberrant behaviors, and social communication within both clinical groups. Exploratory analyses were conducted to examine gender differences in the pattern of correlations for both the FXS and TD groups.

## 3. Results

The demographics and standardized measure scores of all participants in the FXS and TD groups are reported in Table 1. Feasibility was achieved with 100% of participants who attempted ELS-N being able to complete and have adequate utterances for analysis. The two groups did not differ in terms of chronological age (*t*(46) = 0.32, *p* = 0.754) or sex (χ^2^(1) = 0.32, *p* = 0.571). The length of ELS-N recordings was not significantly different between the two groups (*F*(1,47) = 0.27, *p* = 0.612; see Table 2).

### 3.1. ELS Between-Group Analyses

**Figure 1:**
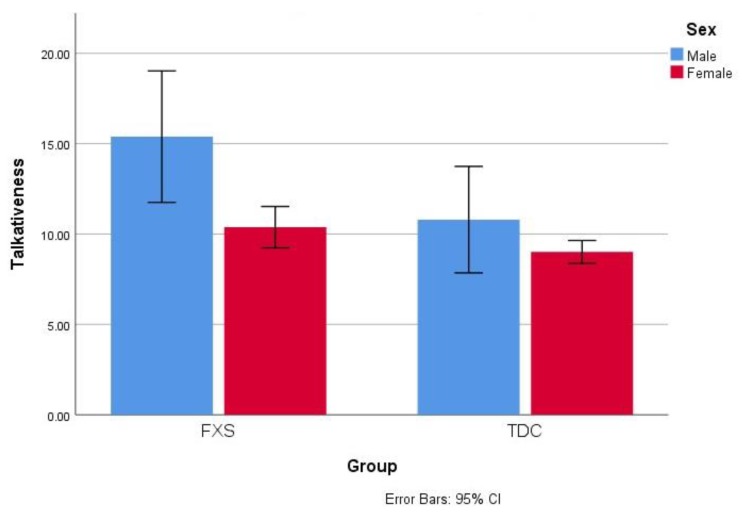
Talkativeness scores are demonstrated for fragile X syndrome (FXS) and typically developing control (TDC) groups and separated by sex. Error bars represent 95% confidence interval.

**Figure 2:**
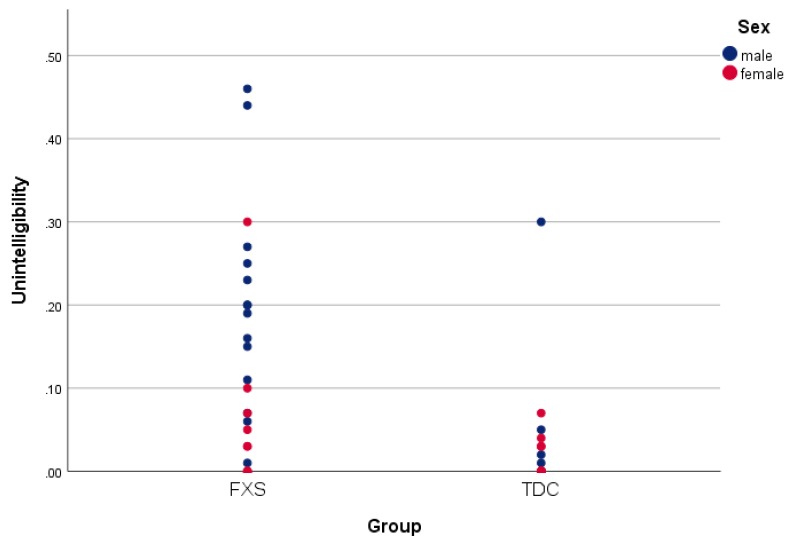
Unintelligibility scores are displayed for both FXS and TDC groups and separated by sex.

For each of the ELS-N variables a significant difference was found between the FXS and TD groups, including a decreased syntactic complexity in FXS (*F*(1,47) = 35.64, *p* = 0.000), a decreased lexical diversity in FXS (*F*(1,47) = 14.12, *p* = 0.000), a worse dysfluency in FXS (*F*(1, 47) = 5.53, *p* = 0.023), an increased talkativeness in FXS (*F*(1,47) = 6.34, *p* = 0.015; see Figure 1), and an increased unintelligibility in FXS (*F*(1,47) = 12.75, *p* = 0.001; see Figure 2 and Table 2).

### 3.2. ELS-N Within-Group Sex Analyses

For the ELS-N variables, no difference was found between FXS males and FXS females for dysfluency, syntactic complexity, or lexical diversity. There was a significant difference between males and females in unintelligibility (*F*(1,24) = 9.04, *p* = 0.006; see Figure 2) and talkativeness (*F*(1,24) = 7.78, *p* = 0.011; see Figure 1), with FXS males being more talkative and less intelligible than FXS females. No within-group differences were found for the ELS-N variables for the TD group.

### 3.3. FXS Clinical Measure Sex Difference Analyses

To further understand the sex-based differences within the FXS group, within-group analyses were conducted for the additional measures. As expected, the FXS group differed by sex in terms of cognitive abilities, with the FXS females performing better than the FXS males (*F*(1,23) = 6.39, *p* = 0.019). There were no significant sex differences in adaptive functioning. For the ADAMS, males and females differed significantly on the manic/hyperactivity scale (*F*(1,21) = 5.63, *p* = 0.28) and generalized anxiety (*F*(1,21) = 4.23, *p* = 0.05), with males having more severe impairments on each. No other ADAMS subscale had significant differences between males and females. A significant difference was found between the sexes on the SCQ (*F*(1,20) = 8.97, *p* = 0.008,) with males having more impairment. On the ABC, a significant difference was found in hyperactivity (*F*(1,19) = 7.91, *p* = 0.012) and inappropriate speech (*F*(1,19) = 13.37, *p* = 0.002), with males having more severe impairments. There were no differences between males and females on ABC irritability, lethargy, or stereotypy.

### 3.4. Correlation Analyses

#### 3.4.1. Cognitive Abilities

With regard to cognitive abilities, a significant correlation was found for the FXS group between higher SB5 ABIQ and increased lexical diversity (*r*(24) = 0.57, *p* = 0.004), increased syntactic complexity (*r*(24) = 0.69, *p* = 0.000), and lower unintelligibility (*r*(24) = −0.62, *p* = 0.001). Considering the two domains that comprise the SB5 ABIQ separately, the results indicate that the SB5 verbal knowledge z-score was significantly correlated with improved lexical diversity (*r*(24) = 0.47, *p* = 0.022), improved syntactic complexity (*r*(24) = 0.62, *p* = 0.001), and decreased unintelligibility (*r*(24) = −0.65, *p* = 0.001) for the FXS group. A similar profile was found for the SB5 nonverbal fluid reasoning z-score, which was correlated with improved lexical diversity (*r*(24) = 0.59, *p* = 0.003), improved syntactic complexity (*r*(24) = 0.69, *p* = 0.000) and reduced unintelligibility (*r*(24) = −0.54, *p* = 0.007). For the TD group, significant positive correlations were found between the verbal knowledge subscale z-score of the SB5 and both lexical diversity (*r*(23) = 0.59, *p* = 0.003) and syntactic complexity (*r*(23) = 0.46, *p* = 0.028).

Within the FXS group for males, increased lexical diversity (*r*(13) = 0.75, *p* = 0.003) and syntactic complexity (*r*(13) = 0.78, *p* = 0.002) were related to higher ABIQ and higher nonverbal fluid reasoning *z*-score (*r*(13) = 0.79, *p* = 0.001 and *r*(13) = 0.82, *p* = 0.001). Increased unintelligibility was related to lower verbal knowledge z-scores in males (*r*(13) = −0.61, *p* = 0.028). The FXS females had a positive correlation between higher verbal knowledge z-scores and increased syntactic complexity (*r*(11) = 0.65, *p* = 0.032). For the TD males, a significant relationship was found between increased lexical diversity and higher verbal knowledge *z*-scores on the SB5 (*r*(11) = 0.71, *p* = 0.014). For the TD females, there were no significant correlations.

#### 3.4.2. Clinical/Behavioral Concerns

For the FXS group, increased talkativeness was correlated with increased symptoms on the ADAMS Compulsive Behavior Scale (*r*(22) = 0.44, *p* = 0.041), ABC Inappropriate Speech (*r*(20) = 0.54, *p* = 0.014), ABC Hyperactivity scales (*r*(20) = 0.45, *p* = 0.047), and higher scores on the SCQ (*r*(21) = 0.49, *p* = 0.025). Further, higher scores on the SCQ was correlated with increased unintelligibility (*r*(21) = 0.67, *p* = 0.001). No significant correlations were found for the TD group.

For FXS females, there was a significant relationship between lower unintelligibility and lower social communication difficulties on the SCQ (*r*(10) = 0.69, *p* = 0.028). No significant correlations were found for the FXS male group. For the TD males, there was a significant relationship between decreased manic/hyperactivity symptoms on the ADAMS and increased syntactic complexity (*r*(11) = −0.63, *p* = 0.039). No significant correlations were found for the TD female group.

#### 3.4.3. Adaptive Skills

Adaptively in the FXS group, lower unintelligibility was associated with better scores on the overall Vineland Adaptive Behavior standard score (*r*(20) = −0.52, *p* = 0.018) and Daily Living Domain standard score (*r*(20) = −0.67, *p* = 0.001). At a subscale level, higher expressive communication v-scores on the Vineland was positively correlated with improved syntactic complexity (*r*(20) = 0.54, *p* = 0.015) and negatively correlated with unintelligibility (*r*(20) = −0.62, *p* = 0.004). Better written expression v-scores was also correlated with better syntactic complexity (*r*(20) = 0.47, *p* = 0.038).

When separated by sex, there was a significant relationship for females between increased syntactic complexity and higher standard scores on the Vineland Expressive Communication subscale (*r*(7) = 0.85, *p* = 0.017), Daily Living Skills Domain (*r*(7) = 0.85, *p* = 0.014), Socialization Domain (*r*(7) = 0.84, *p* = 0.019), and overall adaptive behavior (*r*(7) = 0.85, *p* = 0.016). In addition, greater lexical diversity was related to better daily living skills standard scores for females (*r*(7) = 0.79, *p* = 0.036). There were no significant correlations between ELS-N variables and Vineland standard scores for the male group.

## 4. Discussion

Expressive language deficits are a core component of FXS and a targeted interest in clinical trials, creating the need for valid and reliable measures to appropriately measure change in this domain [6]. Standardized measures tend to inadequately measure expressive language in FXS due to floor effects on standard scores. This clear need led to the development of the ELS-N, a language sampling procedure that has been standardized and used with individuals with FXS, DS, and typical development [5,7,8]. The current study sought to further explore construct validity of the ELS-N by comparing individuals with FXS and TD same-aged peers. In addition, we examined the relationship between ELS-N measures and standardized assessments and rating scales to further explore construct validity. We found that 100% of the FXS and TD groups were able to complete the measure. All ELS-N variables differentiated the FXS group from the TD group, with differences in unintelligibility of speech, syntactic complexity, and lexical diversity providing the most pronounced differences. Although it was expected that the two groups would differ, this had not yet been evaluated and these results further validate the use of ELS-N with both TD individuals and individuals with FXS. In addition, the unique profile of FXS expressive language, as measured by ELS-N in relation to the TD group, is consistent with past research demonstrating differences between FXS, DS, and mental age matched control groups. Additionally, exploratory results provide further construct and discriminant validity for ELS-N through relationships between ELS-N variables and other clinical measures as detailed in the following paragraphs.

Upon further exploration of the two groups separately, interesting relationships emerged between standardized measures and the ELS-N variables. Greater intellectual functioning standard scores, estimated by the Stanford-Binet 5 ABIQ, were associated with greater lexical diversity, syntactic complexity, and intelligibility for the FXS group. In addition, verbal knowledge and nonverbal fluid reasoning standard scores were positively related to syntactic complexity and negatively related to unintelligibility. The stronger one’s nonverbal reasoning abilities were relative to chronological age expectations, the more complex their language and the more intelligible their speech. Our finding extends previous research of a relationship between nonverbal IQ and syntactic complexity [5]. When correlations were completed for FXS males and females separately, it became clear that the relationship between Abbreviated IQ and ELS-N variables were primarily driven by the males in the group. This may be due in part to the larger range of scores on the SB-5 and ELS-N for the male group.

Our finding of a relationship between ASD symptomatology, as measured by the SCQ, and unintelligibility of speech replicates previous findings with a different measure of ASD symptomatology [5]. However, we extend this finding by demonstrating that the relationship with autism symptoms and unintelligibility was only significant for FXS females, not FXS males. Though this finding is a bit unexpected, it may be that unintelligibility in FXS males is prominent regardless of ASD symptoms, whereas only present in FXS females that also demonstrate ASD features. Finally, increased talkativeness was related to more severe compulsive and hyperactive behavior as well as inappropriate speech in the FXS group. In FXS, perseverative language is very common and presents as inappropriate and compulsive utterances as well repetitive topic use. It is, thus, likely that the talkativeness measure on ELS-N is capturing, in part, this phenotypic specific characteristic of FXS. The previous literature has not specifically examined the relationship between talkativeness and clinical measures and this finding should be further explored in future studies as it potentially provides construct validity of the talkativeness scale on ELS-N.

Stronger adaptive skills and daily living skills standard scores unsurprisingly, were related to more intelligible speech. It is likely that individuals with more intelligible speech tend to be higher functioning in general, leading to better overall adaptive skills. Interestingly, standard scores on the Vineland Communication domain was not related to any of the ELS-N variables. This is a bit unexpected but may suggest that ELS-N captures unique aspects of language in FXS that the Vineland Communication standard score does not adequately assess. In addition, it measures a wide range of communication forms, including receptive and written, two scales we would not expect to be related to ELS variables, although expressive would be expected to be related. We subsequently completed follow-up analyses assessing the relationship between ELS-N variables and individual subscale v-scores. We found that the expressive communication v-score was related to the complexity of utterances and unintelligibility of speech; the written expression v-score was also related to syntactic complexity of utterances. This is consistent with past research that found a relationship between expressive and written communication and complexity of utterances [7]. Furthermore, when males and females were separately analyzed, the relationships between ELS-N and Vineland Expressive communication and daily living skills standard scores appeared to be primarily driven by females in the sample. Again, this suggests that whereas characteristics of language tend to co-vary strongly with other measures of functioning in females with FXS, specific language impairments in males with FXS may be more pervasive and present, regardless of more general adaptive functioning. It is also possible that floor effects occurred with the males and not with the females. However, we did find a relationship between higher cognitive function, relative to chronological age expectations, and increased lexical diversity and syntactic complexity for males only. This suggests that more complex and diverse language in FXS males is likely more common among those who are less cognitively impaired relative to chronological age expectations. Of course, this makes sense developmentally, as those that are less cognitively impaired are likely going to be less delayed and better able to learn and adapt new words and grammar into full sentences.

In an effort to further understand the differences between males and females in the FXS group, we examined sex-related differences on each of the clinical measures. Limited empirical data are available on how males and females differ in terms of clinical measures. Interestingly, there were no differences between males and females in terms of adaptive behavior skills standard scores. It is possible that floor effects limited our ability to differentiate between the two sexes. Nonetheless, the males with FXS were significantly more impaired in the areas of cognitive standard scores, ASD symptomatology, hyperactivity (on two separate measures), and generalized anxiety.

When clinical relationships were explored with the TD group, we found a relationship between increased verbal knowledge standard scores and greater lexical diversity and syntactic complexity. This indicates that those individuals with stronger verbal knowledge, relative to chronological age expectations, used both a more diverse vocabulary and longer, more complex communication units. Interestingly, this is a slightly different finding than the FXS group (i.e., both verbal and nonverbal standard scores related to lexical diversity and syntactic complexity). In fact, this idea is supported by findings from a recently published study using continuous EEG recording during speech production in FXS and TD controls [15]. In the TD controls, greater lexical diversity and syntactic complexity related to greater neural activity in frontal and temporal lobes prior to speech onset; a finding not replicated in FXS. Instead, greater high-frequency neural “noise” prior to speech production related to more dysfluent speech among individuals with FXS.

### Limitations

Although the inclusion of a same-aged comparison group was a novel expansion for the study, this study would have been strengthened by the inclusion of a mental age-matched group along with TD and FXS. This would have allowed for in depth analyses about the impact of intellectual ability on the between-group differences. Additionally, future studies would benefit from a broader range of verbal and nonverbal intelligence subscales to better understand relationships between cognitive functioning and ELS-N variables. Though we confirm and extend previous findings on language impairments in FXS using the ELS-N, suggesting its promise as a potential outcome measure in clinical trials, because we only collected at one time point for this study, test-retest data will be important for future studies. Finally, although our sample size was comparable to other ELS-N studies, it is a small sample and future studies would benefit from a larger sample size.

## 5. Conclusions and Future Directions

This study adds to the growing research evidence supporting the use of ELS-N as an outcome measure for individuals with FXS. It is feasible to administer and complete with individuals with a wide range of functioning, as evidenced by the completion rates of the FXS and TD groups. The unique profile of ELS-N variables in relation to intelligence scales suggests that the ELS-N may capture a different set of language processes than those seen in TD individuals. Additionally, ELS-N appears to capture unique aspects of phenotypes among FXS males and females, suggesting its utility as a potential biomarker that it may be more sensitive to clinical change than parent-report measures. Future studies should examine the relationship between ELS-N variables and CGG repeat numbers, seek to replicate the relationships between ELS-N and the clinical measures included in this study, particularly with measures of hyperactivity and talkativeness, and examine ELS-N in a larger sample size.

## Figures and Tables

**Table 1:** Demographic and measure means.

	Fragile X Syndrome	Typical Controls
Sex	56% male	47.8% male
Age	19.96 (8.47)	19.19 (8.43)
SB5 Deviation ABIQ	−3.28 (1.94)	0.098 (0.51)
SB5 Verbal Knowledge z-score	2.13 (5.01)	9.97 (2.02)
SB5 Nonverbal Fluid Reasoning z-score	−1.79 (7.30)	10.61 (2.06)
SCQ	12.86 (7.94)	2.09 (2.17)
ABC Irritability	9.55 (9.92)	0.61 (1.66)
ABC Withdrawal	6.85 (6.98)	1.57 (3.51)
ABC Stereotypy	2.40 (3.09)	0.09 (0.42)
ABC Hyperactivity	10.55 (10.30)	1.39 (3.54)
ABC Inappropriate Speech	4.20 (3.30)	0.17 (0.65)
Adams Manic/Hyperactive	6.00 (3.940)	0.91 (1.70)
Adams Depressed Mood	2.91 (3.64)	1.22 (2.13)
Adams Social Avoidance	7.27 (5.84)	1.30 (2.27)
Adams General Anxiety	6.91 (4.87)	1.96 (2.44)
Adams Compulsive Behavior	2.09 (2.74)	0.30 (0.88)
Vineland Communication Vineland Receptive Communication v-score Vineland Express Communication V-score Vineland Written Communication V-score	51.20 (20.17) 8.25 (2.49) 7.10 (2.49) 6.85 (2.62)	− − − −
Vineland Daily Living Skills	62.05 (12.96)	−
Vineland Socialization	63.20 (11.03)	−
Vineland Adaptive Behavior	57.40 (13.20)	−

**Table 2:** Expressive language sampling (ELS) between-group analyses.

	Fragile X Syndrome	Typical Controls	ANOVA Values	*p* Values
Recording time in minutes	4.68 (1.84)	4.41 (1.66)	0.27	0.612
Syntactic Complexity	6.74 (2.59)	11.51 (3.01)	35.64	0.000
Lexical Diversity	87.96 (40.53)	134.83 (49.23)	14.12	0.000
Dysfluency	20.5 (12.9)%	31.7 (19.3)%	5.53	0.023
Unintelligibility	13.5 (13.3)%	2.4 (6.3)%	12.75	0.001
Talkativeness	13.18 (5.40)	9.86 (3.17)	6.34	0.015
